# Predicting Acute Kidney Injury in Acute Rhabdomyolysis

**DOI:** 10.3390/jcm14196892

**Published:** 2025-09-29

**Authors:** Andy K. H. Lim

**Affiliations:** 1 Department of General Medicine, Monash Health, Clayton, VIC 3168, Australia; andy.lim@monash.edu; 2 Department of Nephrology, Monash Health, Clayton, VIC 3168, Australia; 3 Department of Medicine, Monash University School of Clinical Sciences at Monash Health, Clayton, VIC 3168, Australia

**Keywords:** rhabdomyolysis, creatine kinase, acute kidney injury, renal replacement therapy, prediction, statistical model, epidemiology

## Abstract

Rhabdomyolysis is a clinical syndrome of significant skeletal muscle damage leading to electrolyte disturbance and kidney toxicity, which may result in acute kidney injury. The short-term impact of acute kidney injury includes a severity-dependent increased mortality and need for renal replacement therapy, while long-term effects include development and progression of chronic kidney disease, and increased cardiovascular risk. The ability to predict acute kidney injury early in the presentation is valuable for providing tailored preventative strategies, planning the intensity of monitoring, and appropriate resource allocation. Several clinical variables and biomarkers are reported to be associated with rhabdomyolysis-associated acute kidney injury, and a number of prediction models have been developed for this purpose. However, heterogeneity in study populations and methodology poses challenges to the utility and clinical integration of these variables and prediction models. This article explores and summarizes some of the relevant variables and models used to predict acute kidney injury in rhabdomyolysis, and discusses the uncertainties around the traditional biomarkers like creatine kinase and myoglobin, along with insights from recent observational studies.

## 1. Introduction

Rhabdomyolysis is a clinical syndrome of significant muscle injury that results in the release of the cellular content of muscle cells into the bloodstream, which can be toxic to other cells and cause serious homeostatic imbalance. One of the grave consequences of severe rhabdomyolysis is the development of acute kidney injury (AKI), while the associated electrolyte disturbances include hyperphosphatemia, hypocalcemia, hyperkalemia, hyperuricemia, and metabolic acidosis. These imbalances contribute to the complications of rhabdomyolysis such as cardiac and neuromuscular instability. The typical symptoms of severe rhabdomyolysis include muscle pain and swelling, weakness, discolored urine due to excretion of myoglobin, and poor urine output. However, symptoms are variable, and a full-hand is not universal.

Muscle damage causes a rise in serum creatine kinase (CK), a muscle enzyme involved in energy production within skeletal muscle, cardiac muscle cells and brain tissue. CK is a sensitive but non-specific biomarker for rhabdomyolysis, thus a CK > 5 times the upper limit of normal (>1000 IU/L) is the recommended minimum threshold to confirm rhabdomyolysis. This definition varies between studies, and 5000 U/L is commonly used to define “severe” rhabdomyolysis in some studies. There are many causes of rhabdomyolysis such as trauma and pressure injury, exercise and overexertion, drugs and illicit substances, infection and sepsis, severe electrolyte and metabolic disorders, inherited and genetic disorders, temperature extremes and electrocution, and seizures. The relative frequency of these etiologies depend on the population studied, and combination etiologies are common.

The ability to predict AKI early in rhabdomyolysis may greatly influence the clinical outcome. In recent years, a number of prediction models, biomarkers and scoring variables have been studied in an attempt to provide risk stratification for AKI in patients with rhabdomyolysis. Despite these efforts, early identification of patients at high risk of developing AKI remains challenging. The aim of this article is to discuss current predictive methods and the variables used for AKI prediction in rhabdomyolysis, outline some controversies and difficulties associated with developing accurate prediction models, reasons for imprecision, and to share recent learnings and insights, in the hope of providing suggestions for future research and development.

## 2. Acute Kidney Injury

**Figure 1:**
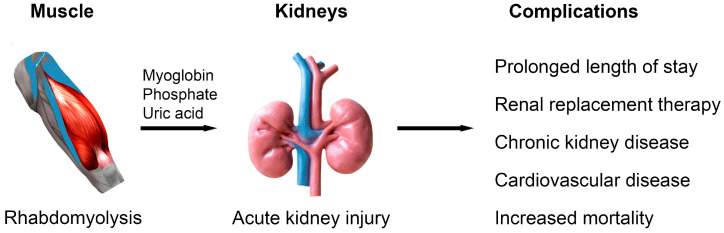
The development of acute kidney injury in patients with rhabdomyolysis is associated with a significant impact on acute and long-term morbidity, mortality, and treatment costs.

The pathogenesis of AKI in rhabdomyolysis is multifactorial but at the core of most cases, myoglobin released from damaged muscle cells cause toxicity to renal tubular cells, obstruction of tubular flow by myoglobin casts and Ca^2+^. PO_4_^−^ deposits, and vasoconstriction of the intrarenal blood vessels [1]. Myoglobin is a cytoplasmic protein required for oxygen storage and utilization. When released into the circulation, myoglobin is freely filtered by the glomeruli and precipitate into obstructive casts. The heme component of myoglobin promotes the formation of reactive oxygen species, which cause oxidative damage to tubular epithelial cells. Volume depletion due to muscle edema and third-space loss result in activation of the renin-angiotensin system, in addition to vasoconstriction due to activation of local vasoactive mediators by myoglobin, lead to renal hypoperfusion [2]. These mechanisms collectively result in acute tubular necrosis. A detailed description of the molecular mechanisms of AKI can be found elsewhere [3]. The development of AKI is significant because AKI is associated with short and long-term consequences (Figure 1). AKI prolongs hospitalization, increases morbidity and cost of hospital stay in association with the need for renal replacement therapy (RRT), and heightens the risk of death as development of AKI perpetuates and worsens electrolyte and metabolic abnormalities, increasing cardiovascular instability. Longer term, patients with AKI have greater risk of developing chronic kidney disease (CKD) or progression of existing CKD to end-stage kidney disease, along with increased cardiovascular disease and death [4,5,6,7].

Therefore, there is a need to predict AKI, primarily because AKI is often preventable or reversible if identified early [8], and tailored preventative strategies may include aggressive fluid resuscitation, avoidance of nephrotoxins, critical care unit admission, intensive monitoring (frequent biochemistry and hemodynamic monitoring, catheterization for urinary flow measurement). Thus, AKI prediction can guide clinical decision making and resource allocation, avoiding unnecessary testing and interventions in low-risk patients and therefore improve patient flow and reduce hospitalization costs.

For the diagnosis of AKI, recent studies use the definition recommended by the Kidney Disease Improving Global Outcomes (KDIGO) group [9]. The KDIGO 2012 definition is based on changes in serum creatinine from baseline and urine output, and divided into 3 stages of increasing severity (Table 1). Many studies only use the creatinine criteria as determination of urine output for all patients is difficult to achieve, and usually not possible in retrospective studies and non-intensive care unit (ICU) settings, while determining baseline creatinine values can pose a challenge as well [10].

## 3. Traditional Biomarkers in AKI Prediction

Traditional biomarkers are typically measured by standard laboratory tests, and should be relatively easy to obtain and interpret. They may be either (1) cellular content released directly from injured muscle cells, such as CK, myoglobin, phosphate (PO_4_^−^), potassium (K^+^), uric acid; or (2) indirect measures of the extent of muscle injury, using surrogates biomarkers measuring the metabolic and physiological disturbance associated with severe tissue injury, such as metabolic acidosis, calcium (Ca^2+^), albumin, inflammatory markers, and coagulation activity. Although indirect biomarkers may be less specific for muscle injury, they are still relevant in AKI prediction as observable risk factors.

### 3.1. Creatine Kinase

An elevated CK is not just essential for confirming rhabdomyolysis but may be useful in predicting AKI, although the correlation is not perfect. The issues with CK as a predictor relate to (1) timing of CK measurement relative to the peak CK level, (2) timing of CK measurement relative to rise in creatinine, (3) CK threshold which conveys risk, (4) functional form of CK in modelling, given its highly skewed distribution and the possibility of non-linear association with risk. Clearance of CK from the circulation is mostly through hepatic and reticuloendothelial systems, with a half-life of approximately 12 h, and is prolonged with chronic liver disease [11]. In observational studies, the elimination half-life was reported to be as long as 36 h, but these estimates may be confounded by ongoing CK generation and appear greater than estimates derived from experimental conditions. Thus, CK may be relatively low depending on the timing of presentation relative to injury. Most studies suggest that the peak CK better predicts AKI than the initial CK at presentation. Simpson et al. noted that in 91% of patients, the peak occurred between admission and day 3 [12], which is similar to our clinical observations.

Clinicians often ask if there is a threshold which confers greater AKI risk and whether risk increases in a linear fashion with CK. Some studies have used a categorical approach to model risk, using a binary category such as CK > 10,000 U/L, or ordinal categories such as 1000–5000 U/L, 5001–15,000 U/L, >15,000 U/L [13]. Others have modelled CK as a continuous variable. In our own prediction models, we found that the log transformed CK provided better model fit compared to modelling with raw CK values as a continuous variable (Table 2).

El-Abdellati et al. also modelled log-transformed CK and demonstrated a non-linear relationship between CK and AKI risk [15]. McMahon et al. found a non-linear association between CK and their composite outcome of RRT or inpatient death, with an increased risk of the composite outcome at CK levels > 40,000 U/L [16]. Candela et al. found a 3-fold higher mean CK in patients with Stage 2–3 AKI compared to patients with Stage 1 or no AKI, but CK was not a statistically significant predictor in their multivariable model as a linear variable [17]. Many studies reported that CK levels are higher in patients who develop AKI compared to patients who did not, but struggle to quantify this relationship because the distribution of CK is highly skewed and the association with AKI/RRT is non-linear. A 2016 systematic review of observational studies of CK for prediction of AKI in patients with rhabdomyolysis concluded that the performance of CK is better for patients with trauma/crush injury compared to non-traumatic etiology [18]. There was high heterogeneity between studies, including the use of old and inconsistent definitions of AKI which limits the interpretation of the meta-analysis. The use of CK as a predictor in a cohort with heterogenous etiologies may be one reason CK does not perform well as a single predictor. Although there is a clear correlation between peak CK levels and AKI risk, a CK level in isolation seems insufficient for optimal risk prediction.

### 3.2. Myoglobin

Myoglobin has a half-life of 2–4 h and peaks much earlier than CK in rhabdomyolysis [15]. Several studies suggest that it is a better predictor of AKI than CK. In trauma and crush injury, peak serum myoglobin was a better predictor of AKI than CK as a single predictor, in cohorts of relatively young patients [19,20]. Raju et al. reported an AUC of 0.78, and at a myoglobin threshold of 5160 ng/mL, there was 79% sensitivity and 79% specificity for predicting AKI. In patients admitted to ICU with severe rhabdomyolysis, Candela et al. found that serum myoglobin at a threshold of 8000 ng/mL was an independent predictor of Stage 2–3 AKI, when CK modelled as a linear variable was not statistically significant [17]. In another large ICU study, El-Abdellati et al. noted that AKI prediction based on serum myoglobin (threshold of 368 ng/mL) provided the best AUC compared with urinary myoglobin or CK [15]. In an ICU study of patients with rhabdomyolysis due to heat stroke, Wu et al. reported that serum myoglobin outperformed CK in AKI prediction (AUC, 0.79 vs. 0.60), and a serum myoglobin ≥ 1000 ng/mL had a sensitivity of 79% and specificity of 68% for predicting AKI [21].

Vangstad et al. further proposed using the myoglobin/CK ratio for risk prediction, demonstrating that the upper third and fourth quartiles of the ratio were associated with an odds ratio of 4.1 and 6.0, respectively, for developing AKI, compared to the 1st quartile. The authors also demonstrated that CK and myoglobin were not independent predictors, and in a multivariable model, CK was not statistically significant while myoglobin retained its strong association with AKI [22]. Thus, it is unnecessary to include CK and myoglobin in the same prediction model unless the myoglobin/CK ratio is used.

The has been no evidence to indicate that urine myoglobin is a useful predictor of AKI in rhabdomyolysis [23], but given the generally supportive observational data for serum myoglobin, why is it not used more frequently in risk prediction? Some of the challenges include the lack of laboratory standardization of assays and the highly variable reported thresholds for risk prediction, availability of assays, and the long turnaround time for test results. Due to assay variability, myoglobin-based prediction models may only have internal validity and not external validity. Point of care tests for myoglobin are available but they are yet to be formally studied in AKI risk prediction.

### 3.3. Alanine Aminotransferase and Aspartate Aminotransferase

Alanine aminotransferase (ALT) and aspartate aminotransferase (AST) are frequently elevated in rhabdomyolysis, raising concern of liver injury. However, muscle release of aminotransferases also explains the association between elevated CK and AST/ALT, as there is no correlation between CK and other liver biomarkers (bilirubin, alkaline phosphatase, γ-glutamyl transferase) [24,25]. Furthermore, while examining the relationship between peak CK and ALT, we noted that peak ALT was also associated with AKI [26]. In that study, we used peak CK as a surrogate marker for the severity of rhabdomyolysis, and demonstrated a strong linear relationship between the log peak CK and log peak ALT. Our results were validated by Chandel et al., who similarly used linear regression to model the association between log CK and log AST in patients with rhabdomyolysis (coefficient of determination, R^2^ = 0.80). Using their model based on AST to predict a CK level ≥ 5000 U/L, they reported an AUC of 0.96. At a threshold AST of 110 U/L, the sensitivity was 97% and specificity was 86%, for detecting CK levels ≥ 5000 U/L [27].

**Figure 2:**
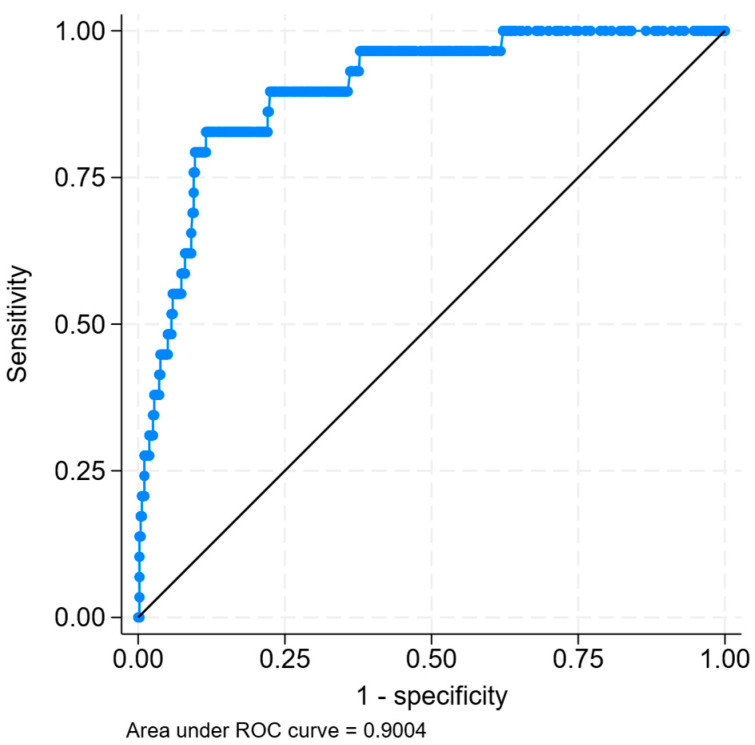
Area under the receiver operating curve of 0.90 for predicting renal replacement therapy in patients with rhabdomyolysis, using the covariates of log-alanine aminotransferase, sepsis syndrome, hyperkalemia, hypocalcemia, and advanced chronic kidney disease.

Akmal and Massry reported that in patients with rhabdomyolysis, higher levels of ALT/AST was associated with AKI [28]. Similarly, Rodriguez et al. reported that patients with AKI were more likely to have an ALT ≥ 259 U/L (63% vs. 33%, *p* = 0.001) or AST ≥ 95 U/L (59% vs. 32%, *p* = 0.008). Given the excellent correlation between CK and ALT/AST, this raises the question as to whether ALT/AST can replace CK as a biomarker in predicting AKI in rhabdomyolysis. Based on data from our previously published study [26], we could also build a prediction model for RRT based on ALT (instead of CK), which included other significant covariates (Table 3). This model demonstrated an excellent area under the receiver operating curve (AUC) of 0.90 for predicting RRT (Figure 2). It should be noted that this model is only valid for patients with a clinical syndrome compatible with rhabdomyolysis and should not be generalized to undifferentiated patients with abnormal aminotransferases. It is also interesting to note that Liu et al. developed a prediction model for AKI which included AST and CK, as both were identified via LASSO regression to be predictive of RRT in the ICU setting [29]. It remains to be established whether both variables are necessary given they are well correlated.

### 3.4. Lactate Dehydrogenase, Aldolase, Carbonic Anhydrase III

Lactate dehydrogenase (LDH) is a cytoplasmic enzyme which converts lactate to pyruvate. LDH is ubiquitous in most cells, but high concentrations are found in muscle, liver, kidneys, and red blood cells. LDH 5 is the predominant isoform in skeletal muscle. Aldolase is a glycolytic enzyme important for energy production and has several isoforms. Aldolase A is muscle-specific, with high concentrations in skeletal muscle, while high concentration of aldolase B is found in liver and kidneys, and aldolase C is brain-specific. The kinetics of aldolase and LDH are relatively slow compared to CK and myoglobin, with a slower rise and longer clearance, and are thus less dynamic [30,31]. There are multiple isoforms of carbonic anhydrase III, an enzyme which catalyzes the conversion of carbon hydroxide and water to bicarbonate and hydrogen. Carbonic anhydrase III is the isoform mainly located in skeletal muscle but is also located in cardiac and smooth muscle, adipocytes, and liver. The kinetics of carbonic anhydrase III is more rapid and similar to that of myoglobin [32]. Although these are potential biomarkers of muscle injury, they have not been studied as predictors of AKI with the exception of LDH. One study suggested predictive value of LDH in combination with myoglobin in predicting AKI in patients with heatstroke [33]. Furthermore, assays which do not measure the muscle-specific isoforms lack specificity. Aldolase and carbonic anhydrase III are not standard laboratory tests and their reference ranges are not standardized, but they have existed for a long time and are not considered novel.

### 3.5. Metabolic Acidosis and Lactate

Several studies identify metabolic acidosis as predictive of AKI in rhabdomyolysis, using a variety of surrogate markers such as blood pH < 7.35 [34], HCO_3_^−^ < 19 mmol/L [16], or lactate > 2.25 mmol/L [35]. In one of their multivariable models, Candela et al. reported a hazard ratio of 0.92 (95% CI: 0.86–0.99) for developing Stage 2–3 AKI for every 1 mmol/L increase in serum HCO_3_^−^ [17], which is interpreted as a higher risk of AKI with lower HCO_3_^−^. According to Seo et al., a lactate > 2.25 mmol/L was associated with an adjusted odds ratio (OR) for AKI of 2.35 (95% CI: 1.08–5.10) in their multivariable model. There is biological plausibility for this observation. Solubility of myoglobin is reduced in acidic environments, so it is more likely to precipitate in kidney tubules and form casts when pH or HCO_3_^−^ (as a proxy for acidosis) is low. A high serum lactate may be a marker of tissue hypoperfusion and also a cause of metabolic acidosis. Fang et al. reported that severe and persistently elevated lactate in the initial 48 h in any patient admitted to ICU was an independent risk factor for AKI [36], and Hsu et al. reported that the initial serum lactate was an independent risk factor for AKI in septic patients presenting to the ED [37].

### 3.6. Calcium-Phosphate

Low Ca^2+^ and high PO_4_^−^ are biomarkers of AKI risk in rhabdomyolysis. McMahon et al. reported an increased risk with Ca^2+^ < 7.5 mg/dL (1.87 mmol/L) and PO_4_^−^ > 4.0 mg/dL (1.29 mmol/L), and both these variables are included in their risk prediction score, with a PO_4_^−^ > 5.4 mg/dL (1.74 mmol/L) worth double the risk points [16]. Similarly, Liu et al. noted that albumin, Ca^2+^ and PO_4_^−^ (among the eight variables identified) were predictive of RRT in patients admitted to ICU with rhabdomyolysis [29]. The study by Candela et al. suggested that AKI risk in ICU patients may be stronger for hyperphosphatemia than hypocalcemia, given that they found the hazard ratio for PO_4_^−^ was 2.2 (95% CI: 1.6–4.9) and Ca^2+^ was not statistically significant in their multivariable model [17]. One explanation of this observation is that Ca^2+^ and PO_4_^−^ may be collinear variables, as Ca^2+^ and PO_4_^−^ have a correlated (reciprocal) relationship. During the initial phase of muscle injury, there is influx of Ca^2+^ into myocytes along with leakage of PO_4_^−^ into the circulation, but the sequestered Ca^2+^ is later released back into the circulation after cell membranes break down [38,39]. Theoretically, in delayed presentations, calcium may either be low, normal, or high depending on the timing of the biochemistry. Perhaps Ca^2+^ is a less discriminating marker in delayed presentations, or that PO_4_^−^ is the primary driver of AKI risk, as suggested by cases of PO_4_^−^ nephropathy where Ca^2+^. PO_4_^−^ deposits in renal tubules [40]. Hypocalcemia may be a secondary phenomenon due to Ca^2+^ binding to the PO_4_^−^ released from myocytes. The Ca^2+^ × PO_4_^−^ product could be another consideration, and has been shown to predict AKI and mortality when measured within 24 h of hospital admission [41]. Ca^2+^ × PO_4_^−^ has not been studied as a predictor variable in rhabdomyolysis.

### 3.7. Potassium

There is inconsistency regarding K^+^ as a predictor of AKI risk. Hyperkalemia is probably an indicator of the severity of muscle injury rather than a direct cause of AKI. If so, K^+^ should correlate well with CK levels and PO_4_^−^, unless severe K^+^ deficiency is part of the etiology of rhabdomyolysis. In our study [14], there was a weak correlation between hyperkalemia (K^+^ ≥ 6.0 mmol/L) and log peak CK (*r* = 0.16, *p* < 0.001) and a moderate correlation with hyperphosphatemia (*r* = 0.47, *p* < 0.001). So, it is possible there is collinearity between PO_4_^−^ and K^+^ in multivariable models, but less so with CK. However, Rodriguez et al. noted that hyperkalemia (K^+^ ≥ 5.5 mmol/L) was only a significant predictor of AKI in univariable analysis, but not independent of the covariates of CK, albumin, pH and prothrombin time in their multivariable model [34]. On the other hand, in the ICU setting, Candela et al. reported that K^+^ demonstrated hazard ratio of 1.5 for Stage 2–3 AKI per mmol/L increase (95% CI: 1.1–2.0), independent of PO_4_^−^ which demonstrated a hazard ratio of 2.2 for Stage 2–3 AKI per mmol/L increase (95% CI: 1.6–4.9) [17]. Seo et al. reported a negative association between K^+^ and the risk of AKI, with the upper quartiles of K^+^ demonstrating no increased risk from the first quartile [35]. Thus, K^+^ is a complex variable to include in multivariable models and its interaction with other variables is unpredictable.

### 3.8. Uric Acid

A systematic review suggested that serum uric acid may be a useful predictor of AKI, and the accompanying meta-analysis of six cross-sectional studies demonstrated a standardized mean difference of 1.61 mg/dL (95% CI: 0.69–2.54) in serum uric acid between patients with AKI and patients without AKI [42]. However, the cause of rhabdomyolysis was limited to trauma in the included studies, with three studies including earthquake victims. Thus, the correlation may not be generalized to non-traumatic rhabdomyolysis and limits its use in AKI prediction. Although a higher uric acid level was associated with AKI in trauma and crush injury, the cross-sectional nature of the studies did not allow for causality inference, as patients with AKI also have reduced uric acid excretion.

### 3.9. Markers of Inflammation and Coagulation Cascade

Systemic and renal inflammation play an important role in rhabdomyolysis associated AKI, especially activation of the innate immune system which drives proinflammatory cascades [3]. The extrinsic coagulation pathway is activated by release of tissue factor from damaged muscle cells, resulting in thrombin generation and platelet-fibrin deposition. Inflammatory mediators (e.g., interleukins, C-reactive protein, complement) upregulate kidney expression of tissue factor by local monocyte-macrophages, increase platelet responsiveness and endothelial cell reactivity [43]. Therefore, biomarkers of inflammation and abnormal coagulation may predict AKI in rhabdomyolysis. In patients admitted to ICU following heatstroke and rhabdomyolysis, Wu et al. reported that elevated lymphocyte and neutrophil counts, prothrombin time and D-dimer were variables associated with development of AKI [21]. In patients with trauma-related rhabdomyolysis, Yi et al. noted that D-dimer, C-reactive protein, and platelets were independent predictors of AKI among their list of covariates [44]. However, the effect of prothrombin time on AKI risk is not straightforward. Both Candela et al. [17] and Rodriguez et al. [34] noted that decreased prothrombin time were predictive of AKI development in rhabdomyolysis. Low platelets, prolonged prothrombin time (from consumption of clotting factors), low fibrinogen and elevated D-dimers (from fibrin degradation) are markers typical of disseminated intravascular coagulation (DIC). However, in the initial stages of DIC, there may be circulating activated clotting factors, or that the inflammation driven transient increase in clotting factors may lead to a shortened prothrombin time. The prothrombin time may be normal or shortened in 25–50% of patients with DIC [45], so it remains unclear how prothrombin time should be modelled. Nonetheless, DIC compounds myoglobin-induced injury due to formation of microthrombi in the renal circulation (thrombotic microangiopathy).

## 4. Novel Biomarkers in AKI Prediction

Besides traditional biomarkers, several novel biomarkers may be relevant in AKI prediction in rhabdomyolysis. Some biomarkers are directly related to muscle injury and released by damaged muscle cells, while others are non-specific biomarkers of inflammation and kidney tubular injury, indirectly triggered by the release of injurious agents from muscle or the inflammatory cytokine cascades which follow the initial injury.

### 4.1. Muscle Related Markers

MicroRNAs are non-coding RNA molecules that regulate gene expression by inhibiting the translation of target messenger RNA or promoting mRNA degradation, ultimately acting as post-transcriptional regulators in diverse cellular processes. Increased levels of several muscle specific microRNAs in the circulation have been shown in animal experiments to correlate with direct muscle injury, with peak levels occurring approximately 12 h after injury, and miR-206 and miR-133b is reported to be more specific for skeletal muscle compared to cardiac myocytes [46,47]. In observational studies, miR-1, miR-133, miR-208 and miR-499 were significantly elevated after a 24 h ultramarathon race where participants developed exertional rhabdomyolysis with an average CK of about 50,000 U/L [48]. However, there are no studies yet specifically examining muscle specific microRNA in rhabdomyolysis associated AKI [49]. Experimentally, both fatty acid binding protein 3 (FABP3) and myosin light chain 3 (MYL3) are significantly elevated in muscle injury and demonstrate similar tissue distribution to CK [50]. Urinary excretion of FABP3 predicts RRT in patients with AKI [51,52] but these biomarkers have not been specifically examined in patients with AKI due to rhabdomyolysis.

### 4.2. Kidney Related Markers

Neutrophil Gelatinase-Associated Lipocalin (NGAL) is an early biomarker of AKI, and elevated levels of NGAL showed promise as a predictive tool in general studies of AKI from diverse causes. However, a multicenter prospective study demonstrated that serum NGAL was not a good predictor of AKI in patients with rhabdomyolysis at 48 h after presentation to ED, with a model AUC of 0.64 (95% CI: 0.54–0.74) [53]. Although the study limitations included a low rate of AKI and relatively low peak CK levels, a systematic review also noted no significant correlation between NGAL and AKI in the context of exertional rhabdomyolysis [54]. Kidney Injury Molecule-1 (KIM-1) is a transmembrane protein expressed in proximal renal tubules following injury. Elevated urinary KIM-1 levels have been associated with both the severity of AKI and the risk of progression to renal failure. Interleukin-18 is a pro-inflammatory cytokine that plays a role in the pathophysiology of AKI. Its levels rise early in response to renal injury and may serve as an early indicator of AKI risk. Similarly, FABP3 is also found in kidney tubular cells and is increased early in AKI. To date, KIM-1 and FABP3 have not been specifically examined in patients with rhabdomyolysis. Recently, growth differentiation factor-15 (GDF-15) was used to create the GDF-TRACK-AKI score for predicting AKI in patients with rhabdomyolysis due to exercise or trauma [55]. GDF-15 is cytokine upregulated in cellular stress and inflammation and belongs to the transforming growth factor-β superfamily. Experimentally, GDF-15 levels increase early in renal ischaemia before detectable changes in kidney function [56]. There is currently no evidence that these novel biomarkers are superior to traditional biomarkers or other clinical predictors of AKI.

### 4.3. Biomarkers and Chronic Kidney Disease Risk

Several biomarkers have also proven to be useful in predicting AKI progression to CKD. Data published by the ASSESS-AKI Consortium on a prospective cohort of 656 patients (median age, 65 years) hospitalized with AKI indicated that each standard deviation increase in urine KIM-1, MCP-1 and plasma TNFR1 from baseline to 12 months was associated with a 2- to 3-fold increase in CKD risk after median follow-up of 4.3 years [57]. Another prospective cohort study of 441 patients in the UK examined biomarkers 3 months after hospitalization with AKI, and followed patients for three years. Elevated levels of soluble TNFR1, TNFR2 and NGAL were associated with increased risk of CKD progression during follow-up [58]. Thus, persistent elevation of certain biomarkers can identify patients at risk of progressing to CKD even when traditional markers like serum creatinine have normalized.

## 5. Other Considerations in AKI Prediction

### 5.1. Age and Sex

Several studies indicate that older age is predictive of AKI risk in rhabdomyolysis [16,29,35,44]. However, this is not consistent across all studies. In our study, age was not significant in the multivariable analysis, either for AKI or RRT. In contrast, our initial univariable analysis showed that age was inversely associated with RRT [14], which may be due to treatment bias in elderly patients. Furthermore, we found that age is highly correlated with several variables, including peak CK and comorbidities such as advanced CKD. Thus, age was not statistically significant in our multivariable model and did not improve performance or precision of the model when dropped. Only the McMahon study included patient sex in the risk prediction model [16], which has not been duplicated in other studies. The McMahon study used a composite outcome of RRT/inpatient death, which may have accounted for this unique observation. Analysis of a large US administrative dataset based on a discharge diagnosis of rhabdomyolysis and AKI noted that patients with AKI were more likely to be older and less likely to be female [6]. However, this was not adjusted for comorbidities, and a higher Charlson comorbidity index was also associated with higher risk of AKI. Males often have higher mortality following AKI which may be partly due to greater comorbidities [59]. It is likely that age may not be statistically significant or contribute to performance of a multivariable model if the covariates have accounted for the effect of age on AKI risk.

### 5.2. Etiology of Rhabdomyolysis

Several studies suggest that the cause(s) of rhabdomyolysis is relevant to the risk of AKI or RRT. Melli et al. noted a higher risk of AKI when multiple causes of rhabdomyolysis were implicated compared to a single cause [60]. Similarly, Seo et al. noted that both the etiology and presence of multiple triggers for rhabdomyolysis were associated with higher AKI risk in univariable analysis [35]. McMahon et al. noted that patients with rhabdomyolysis due to seizures, syncope, exercise, statins, or myositis had a lower risk of RRT/inpatient death compared to other causes [16]. With regards to statins, Seo et al. noted a higher risk of AKI with statin use [35], so it remains unclear what risk statin users face. To further this concept, we previously demonstrated that rhabdomyolysis in the context of illicit drug use was associated with a higher risk of AKI and RRT, independent of CK levels [14]. Compared to other patients with rhabdomyolysis, patients with illicit drug use had an adjusted OR for AKI of 2.8 (95% CI: 1.8–4.4), when including covariates of log peak CK, sepsis, cardiovascular disease and pressure ulcer. Only 5% of all patients required RRT, and patients with illicit drug use had an adjusted OR for RRT of 3.2 (95% CI: 1.1–8.9), when including covariates of log peak CK, sepsis, cardiovascular disease, advanced CKD, fasciotomy. Our findings were supported by Yang et al., who reported that opioid and cocaine use among patients with rhabdomyolysis were associated with higher rates of AKI [6]. This risk factor may only be relevant in populations with high prevalence of illicit drug use, such as those reported by Rodriquez et al. [34] and Melli et al. [60].

### 5.3. Concurrent Sepsis

Sepsis is strongly associated with AKI and need for RRT in critically ill patients with rhabdomyolysis, independent of illness severity [61]. We also demonstrated that sepsis was associated with a 3.5-fold higher odds of experiencing a higher stage of AKI and 6.3-fold higher odds of needing RRT, compared to patients without sepsis, independent of peak CK and presence of advanced CKD [14]. Sepsis also interacts with rhabdomyolysis, exacerbating severity of AKI [62]. As sepsis is associated with both rhabdomyolysis and AKI, it represents a confounder which should ideally be accounted for in prediction models. It may be considered as a clinical syndrome in the prediction model, such as using the Sepsis-3 definition [63], or accounted for by including biochemical surrogates of sepsis such as inflammatory markers (discussed in Section 3.9), or the use of vasopressors for hypotension within the ICU setting [17].

### 5.4. Chronic Kidney Disease

Epidemiological studies demonstrate a link between CKD and AKI, which has been described as interconnected syndromes [64]. Meta-analysis have shown that low estimated glomerular filtration rate (eGFR) and albuminuria are strong risk factors for AKI [65]. Seo et al. also noted that CKD was a robust predictor of AKI in patients with rhabdomyolysis presenting to the Emergency Department, with an adjusted odds ratio of 22.6 if baseline creatinine was >1.3 mg/dL (115 µmol/L) [35]. In our rhabdomyolysis study, we noted that advanced CKD (eGFR < 30 mL/min/1.73 m^2^) was an independent risk factor for AKI requiring RRT, with an adjusted odds ratio of 8.2 [14]. Even machine learning models have identified baseline creatinine as a relevant risk factor [44]. Thus, CKD or baseline kidney function warrants consideration in any multivariable AKI prediction model.

## 6. Risk Prediction Models

Despite a plethora of published prediction models, few have progressed to clinical use. This is due to a number of factors: (1) Poor quality studies with small numbers of patients with inconsistent and older definitions of AKI, (2) poor model performance, or lack of reporting of model performance (3) lack of external validation, (4) limited generalizability, due to selective population studied or inclusion/exclusion criteria (e.g., applying models derived from studies of trauma patients to non-trauma cases).

### 6.1. Artificial Intelligence Models

In broad terms, artificial intelligence (AI) approaches such as machine learning (ML) and deep learning (DL) are computer programs designed to predict outcomes based on analysis of complex patterns in data. ML uses algorithms to analyze a training dataset for patterns and relationships which predict the outcome of interest, then make inferences about new data provided. DL advances the ML approach by using neural networks with multiple layers to learn from complex patterns and large data, employing algorithims in a hierchical manner with progressively nuanced interpretation. AI approaches are suited to clinical areas where a standard set of data is routinely collected and updated such as during ICU admission. Information technology advances have allowed AI models to potentially create automated early predictions based on real-time data from clinical and laboratory inputs. ML models such as Extreme Gradient Boosting (XGBoost), Random Forest, Least Absolute Shrinkage and Selection Operator (LASSO), naïve Bayesian, and Support Vector Machines are relatively commonly used for predicting AKI. A comprehensive discussion of AI models is beyond the scope of this article and are reviewed elsewhere [66,67,68,69,70].

Several studies of AI prediction of AKI in rhabdomyolysis have been reported. Liu et al. used LASSO to predict RRT, and identified atrial fibrillation as a novel clinical predictor [29], which was unlikely to have been identified by conventional analysis. Zhang et al. examined a number of ML models to predict rhabdomyolysis-induced AKI using data from the eICU Collaborative Research Database and the Medical Information Mart for Intensive Care III (MIMIC-III) databases. Based on data collected within 24 h of admission, they found that the XGBoost model demonstrated the best performance for predicting AKI. The model identified 10 clinical and 10 laboratory risk factors which included a broad range of variables such as heart rate, mean arterial blood pressure and illness severity scores such as the Sequential Organ Failure Assessment (SOFA) score [71]. Similarly, a XGBoost ML approach was used by Yi et al. to predict AKI in trauma patients with rhabdomyolysis at 24 h and 48 h, which included an injury severity score in addition to 7 other standard predictor variables [44]. Poorsavi et al. used data from earthquake victims with crush and pressure injuries to predict AKI on the third day, applying a multi-layer perceptron neural network approach [72]. Despite these promising studies, model overfitting may be an inherent risk of AI models if no limits are set. The systematic reviews by Shi et al. and Vagliano et al. noted that many AI studies for predicting AKI showed a high risk of bias which potentially affected generalizability and applicability [70,73]. Further work is required to validate AI models and improve clinical integration.

### 6.2. Examples of Risk Prediction Models

Two recently published models are reasonable examples (Table 4). McMahon et al. developed a prediction model based on a retrospective study of 2371 patients in Boston, USA [16]. This model uses a composite outcome of inpatient death/RRT, which clinicians may find difficult to interpret due to competing risks and heterogeneity of effect of potential interventions. However, Simpson et al. validated the applicability of this model in 2016, showing an AUC of 0.78 (95% CI: 0.69–0.87) for prediction of RRT using the McMahon model, and a score ≥ 6 on admission was associated with a sensitivity of 86% and specificity of 68% for AKI requiring RRT, which was superior to peak CK as a single predictor variable [12]. Liu et al. used LASSO regression to develop a prediction model and nomogram in 2024, but it is unclear if the model can be applied outside the ICU setting [29]. Furthermore, no other study has found atrial fibrillation to be an independent risk factor for AKI, and the mechanism for the association remains unclear.

## 7. Limitations of Prediction Models

Prediction models analyze historical data for relationships and create a statistical equation or algorithm to estimate the probability of AKI based on those predictor variables. There are limitations to prediction models worth considering. The data used for model building or training greatly influences the generalizability and performance of the model. Favorable characteristics of a good prediction model is summarized in Table 5.

In applying these prediction models, consider the intended population and setting, and the availability of specific predictors. Some scenarios worth considering include (1) traumatic versus non-traumatic rhabdomyolysis, (2) critical care settings, and (3) resource poor settings. Many studies have been conducted exclusively in patients with traumatic rhabdomyolysis such as earthquake victims with crush syndrome. With a restricted study population, it remains unclear whether predictors and models derived from such studies can be generalized to patients with non-traumatic rhabdomyolysis, or vice versa. Crush injury patients often experience reperfusion injury and compartment syndrome, resulting in a delayed peak or sustained rise in CK and myoglobin, along with other blunt trauma and shock due to hemorrhage. The extrication time and complexity of retrieval may also impact timing of presentation to hospital. In critically ill patients admitted to ICU, many additional factors contribute to risk of AKI, including the impact of vasopressor and inotrope use, mechanical ventilation, sepsis, and exposure to nephrotoxins. Thus, in trauma and critical illness, incorporating an injury or illness severity score is logical and may improve model predictions during ICU admission. Indeed, several studies demonstrated that the SOFA score was a predictor of AKI in patients admitted to ICU with rhabdomyolysis [15,61,71]. In contrast, in resource poor areas, there may be limited access to biomarkers. Clinical variables and basic biochemistry may be the only predictors available. In such cases, a pragmatic model is needed which hopefully remains clinically useful and has acceptable discrimination. Whichever set of variables or model is chosen, it should be validated for clinical use in the local setting and population.

The final point to consider is biomarker sampling, specifically the best timing and need for repeat measures or kinetic profiling. It can be argued that AKI risk is dynamic rather than static, and biomarker kinetic profiling has advantages by determining trends and trajectory, and better identification of peak levels when the time of injury is uncertain or injury is ongoing. Isshiki et al. studied 272 patients admitted to ICU and measured urinary L-FABP on admission and 24 h later. By adding the second L-FAPB measurement to their AKI prediction model including clinical variables and admission L-FABP, the investigators noted a modest improvement in AUC and a marginal improvement in model discrimination [74]. Another ICU study of 156 patients found that the trajectory of biomarkers with serial testing at 1, 12, 24, and 48 h after ICU admission was predictive of major adverse kidney events (composite of death, dialysis dependency, persistent loss of eGFR > 25%). Three basic trajectory patterns were described (low and constant, high and exponential decrease, and high and exponential increase) and the initial level and 12 h change in urine L-FAPB were significantly associated with MAKE [75]. Although these studies were not specific for rhabdomyolysis, they provide proof of concept that serial testing may improve prediction models. We routinely perform serial CK and other laboratory indices, and it makes sense to consider serial measurements of novel biomarkers. However, serial testing is not pragmatic and may not be clinically useful if timely intervention is needed, and such models may have limited generalizability. Nonetheless, studies should ideally specify the timing of biomarker testing for their prediction models, so that external validation can be appropriately conducted based on the specifications of the original model development.

## 8. Conclusions

Predicting AKI in rhabdomyolysis is challenging. The traditional biomarkers of serum myoglobin and CK provide useful information but are insufficient to predict AKI with high accuracy as single variables. Multivariable models appear superior and the parameterization of CK and myoglobin may impact model performance. A prediction model for RRT requirement based on peak liver aminotransferase levels may be an alternative to peak CK levels, when peak CK levels are unreliable or cannot be determined. Markers of illness severity may be relevant in certain situations such as severe trauma, crush injuries and critically ill patients admitted to ICU. Novel biomarkers show promise but barriers to implementation include availability of rapid tests with sufficient standardization to allow external validity. AI systems and predictions have become commonplace, but there is still significant bias among AI prediction studies and there is a tendency to include too many variables which risk model overfitting and poor generalizability. Thus, most pragmatic risk prediction models combine common biochemistry results and clinical information. A combination of clinical judgment, timely biomarker testing, and technological innovations should lead us to more precise prediction models. However, there are many limitations of prediction models for AKI in patients with rhabdomyolysis, and clinicians and researchers should consider their setting and population when considering which predictor variables and models are best suited to their clinical area, taking into consideration model accuracy, pragmatism, clinical usefulness and resources.

## Figures and Tables

**Table 1:** Kidney Disease Improving Global Outcomes (KDIGO) 2012 acute kidney injury staging.

Stage	Serum Creatinine Criteria ^1^	Urine Output Criteria
1	1.5–1.9 × baseline, or ≥0.3 mg/dL increase	<0.5 mL/kg/h for 6–12 h
2	2.0–2.9 × baseline	<0.5 mL/kg/h for ≥12 h
3	3.0 × baseline, or increase to ≥4.0 mg/dL, or RRT	<0.3 mL/kg/h for ≥24 h, or anuria ≥12 h

^1^ Conversion factor, 1 mg/dL = 88.4 µmol/L. Abbreviation: RRT, renal replacement therapy.

**Table 2:** Comparison of RRT prediction model fit based on parameterization of CK (*n* = 641).

	Raw CK	Log CK
Correlation with peak creatinine, *r*	0.161 (*p* < 0.001)	0.250 (*p* < 0.001)
Correlation with change in creatinine, *r*	0.118 (*p* = 0.003)	0.161 (*p* < 0.001)
Logistic regression for RRT, pseudo-R^2^	0.052	0.100
Logistic regression for RRT, Akaike IC	233.6	221.9
Logistic regression for RRT, Bayesian IC	242.6	230.9

Abbreviation: RRT, renal replacement therapy; IC, information criteria; CK creatine kinase. Data from Lau Hing Yim et al. [14].

**Table 3:** Prediction model for renal replacement therapy based on alanine aminotransferase (ALT).

Variable	Odds Ratio	95% C.I.	*p* Value
ALT, per one log U/L increase	2.26	1.48, 3.45	<0.001
Presence of sepsis syndrome	4.67	1.88, 11.5	0.001
Hyperkalemia, potassium > 6.0 mmol/L	3.22	1.25, 8.27	0.016
Hypocalcemia, calcium < 1.80 mmol/L	4.35	1.73, 10.9	0.002
CKD, eGFR < 30 mL/min/1.73 m^2^	7.55	1.33, 43.2	0.023

Abbreviations: CKD, chronic kidney disease; eGFR, estimated glomerular filtration rate.

**Table 4:** Recent examples of risk prediction models for AKI and RRT in adults with rhabdomyolysis.

	McMahon et al. [16]	Liu et al. [29]
Year of publication	2013	2024
Population studied	General hospitalized patients, combination of medical and surgical patients	Patients admitted to multiple intensive care units
CK inclusion threshold	5000 U/L	1000 U/L
Number of patients	Derivation, *n* = 1397 External validation, *n* = 974	Derivation, *n* = 656 Internal validation, *n* = 282 External validation, *n* = 321
Age of patients	Mean 52.4 (SD, 19.7) years	Mean 56.0 (SD, 12.1) years
Top 5 causes of rhabdomyolysis	Trauma (26.3%) Immobilization (18.1%) Sepsis (9.9%) Vascular surgery (8.1%) Cardiac surgery (5.9%)	Trauma (20.0%) Metabolic/electrolyte (15.9%) Infection (12.2%) Alcohol (9.3% Myopathy (8.5%)
Outcome	Composite RRT or death	RRT
AKI definition	KDIGO creatinine criteria	KDIGO criteria
Incidence of AKI	47.7%	71.3%
Incidence of RRT	8.0%	15.9%
Inpatient mortality	14.1%	10.7%
Model variables	***Biochemical:*** Peak CK, initial creatinine, Ca^2+^, PO_4_^−^ HCO_3_^−^ ***Clinical:*** Age, female sex, cause of rhabdomyolysis	***Biochemical:*** Peak CK, baseline creatinine, Ca^2+^, PO_4_^−^, aspartate aminotransferase, albumin ***Clinical:*** Age, atrial fibrillation
Model performance	Derivation, AUC 0.82 External validation, AUC 0.83	Derivation, AUC 0.82 Internal validation, AUC 0.79 External validation, AUC 0.82

Abbreviations: CK, creatine kinase; AUC, area under receiver operating curve; AKI, acute kidney injury; RRT, renal replacement therapy; KDIGO, Kidney Disease Improving Global Outcomes; SD, standard deviation.

**Table 5:** Important characteristics of prediction models for successful implementation.

Characteristic	Description
Discrimination	Correctly identifies patients who develop AKI. May be affected by poor quality or insufficient data, or unrecognized confounders.
Calibration	Good agreement between predicted and observed outcome. Poor calibration may be due to incorrect model specification or assumptions.
Validation	Performs accurately on new data in real-world scenarios. External validation ensures model performs consistently and fit-for-purpose.
Generalizable	Works across different populations and settings. Poor generalizability may be due to selection bias, AKI definitions, and model overfitting.
Pragmatic	Balances need for accuracy with real-world availability of predictor variables which are not routinely available or require special tests.
Parsimonious	Avoid unnecessary complexity and excessive predictors that contribute to model overfitting, poor generalizability or interpretability.
Clinical usefulness	Models can be easily implemented, easy to interpret, and helps with clinical decision making for interventions to prevent AKI.

Abbreviations: AKI, acute kidney injury.

## Data Availability

The data used for analysis to generate Table 2 and Table 3 and Figure 2 was derived from previous published studies, and may be available from the corresponding author upon reasonable request. No new data was generated for this perspective article.

## Abbreviations

The following abbreviations are used in this manuscript (in alphabetical order):

AKI	Acute kidney injury
ALT	Alanine aminotransferase
AI	Artificial intelligence
AST	Aspartate aminotransferase
AUC	Area under receiver operating curve
Ca^2+^	Calcium
CK	Creatine kinase (creatine phosphokinase)
CKD	Chronic kidney disease
DIC	Disseminated intravascular coagulation
DL	Deep learning
eGFR	Estimated glomerular filtration rate
GDF-15	Growth differentiation factor-15
HCO_3_^−^	Bicarbonate
ICU	Intensive care unit
K^+^	Potassium
KIM-1	Kidney injury molecule-1
KDIGO	Kidney Disease Global Outcomes
LASSO	Least Absolute Shrinkage and Selection Operator
LDH	Lactate dehydrogenase
LFT	Liver function test
ML	Machine learning
MAKE	Major adverse kidney events
MCP-1	Monocyte chemoattractant protein-1
NGAL	Neutrophil gelatinase-associated lipocalin
OR	Odds ratio
PO_4_^−^	Phosphate (phosphorus)
RRT	Renal replacement therapy
SOFA	Sequential Organ Failure Assessment
TNFR	Tumor necrosis factor receptor
